# Shea Butter Application to the Umbilical Stump Resulting in Omphalitis and *Leclercia adecarboxylata* Bacteremia in a Neonate: A Case Report

**DOI:** 10.1155/crdi/9889633

**Published:** 2025-12-14

**Authors:** Mayowa Banke Omotosho, Williams Oluwatosin Adefila, Abdulsalam Olawale Yusuf, Isaac Osei, Baleng Mahama Wutor, Momodu Lamin Keita, Molfa Minteh, Ousman Barjo, Muhammed Wally, Rasheed Salaudeen, Grant Mackenzie

**Affiliations:** ^1^ Department of Disease Control & Elimination, Medical Research Council Unit, The Gambia at London School of Hygiene & Tropical Medicine, Banjul, Gambia, mrc.gm; ^2^ Faculty of Infectious and Tropical Diseases, London School of Hygiene & Tropical Medicine, London, UK, lshtm.ac.uk; ^3^ Department of Infection and Immunity, Murdoch Children’s Research Institute, Melbourne, Australia, mcri.edu.au; ^4^ Department of Paediatrics, University of Melbourne, Melbourne, Australia, unimelb.edu.au

**Keywords:** bacteremia, case report, *Leclercia adecarboxylata*, neonate, omphalitis, shea butter

## Abstract

*Leclercia adecarboxylata* is a gram‐negative, rod‐shaped bacterium commonly found in nature. Its pathogenicity is mild and often results in asymptomatic carriage. However, it can cause sepsis and other life‐threatening illnesses in immunocompromised individuals. Based on previous literature, the common practice of applying many unsafe substances, such as shea butter, to the umbilical cord of newborns for healing remains a significant risk factor for omphalitis and neonatal sepsis in developing countries. This case report describes a neonate showing signs of omphalitis and bacteremia, following the use of shea butter on the umbilical cord. A 10‐day‐old male neonate presented with irritability, refusal to breastfeed, and an erythematous, tender umbilical stump. The blood culture result yielded a gram‐negative rod, *Leclercia adecarboxylata*, sensitive to cephalosporin, chloramphenicol, and gentamicin. The newborn was treated with empirical first‐line antibiotics according to national guidelines: intravenous (IV) ampicillin and gentamicin for 6 days. The child fully recovered. Therefore, we emphasize the ongoing need for community‐level awareness of proper umbilical cord care at the grassroots in developing countries. Additionally, early bacterial detection and antimicrobial management based on local antibiogram data are vital for successful patient outcomes.

## 1. Introduction

The dogmatic traditional beliefs surrounding the care of the umbilical stump in many lower‐middle‐income countries (LMICs) continue to pose a significant threat to neonatal lives and well‐being [1]. These cultural practices involve applying unrecommended substances such as shea butter, a fatty material traditionally derived from the nuts of the African shea tree, to the umbilical stump for cord dressing. Meanwhile, the use of such unsterile products on the umbilical cord exposes vulnerable newborns to risks of omphalitis and opportunistic infections [1–3].

*Leclercia adecarboxylata* is a gram‐negative, rod‐shaped bacterium belonging to the prominent family *Enterobacteriaceae*. Before developing more precise DNA‐based methods of bacterial classification*, L*. *adecarboxylata*’s phenotypic similarities to *Escherichia coli* led to its initial designation as *Escherichia adecarboxylata* [4–6]. But in 1987, *L*. *adecarboxylata* was reclassified as a distinct genus and renamed *L. adecarboxylata* [6]. *L. adecarboxylata* is part of the normal flora of the human gastrointestinal tract, is ubiquitous, and has been isolated from various sources, including food, water, soil, and the intestinal tract [7].

Although *L. adecarboxylata* is generally regarded as a rare pathogen, it is not entirely nonpathogenic. It can cause infections, especially in immunocompromised individuals and occasionally in immunocompetent individuals [8, 9]. There have been reports of *L. adecarboxylata* causing invasive diseases in neonates. Myers et al. documented the first case of *L. adecarboxylata* bacteremia in a neonate, linked to previous antibiotic treatments [10]. More recently, the organism has been associated with sepsis in a neonate with Hirschsprung disease [11]. In neonates, the risk factors for *L. adecarboxylata* infection include prematurity, low birth weight, unhygienic birth attendance, maternal mastitis, and immunosuppressive conditions [12]. We report a case of shea butter use for umbilical cord care, which resulted in omphalitis and *L. adecarboxylata* bacteremia in a neonate.

## 2. Case History and Examination

A 10‐day‐old African male neonate was brought to the clinic by his mother with complaints of intermittent fever, refusal to breastfeed, poor suckling, and irritability over the past two days. The mother reported noticing blood spots around the umbilical stump with no visible purulent discharge. She also mentioned applying shea butter to the umbilicus, as it is a common practice for newborn care in rural Gambia. There was no history of vomiting, diarrhea, yellowness of the eyes and skin, or abnormal body movements.

The pregnancy was booked and uneventful, and the mother regularly attended antenatal care (ANC) visits. The pregnancy was a twin gestation, and both babies were carried to term. The mother had no significant previous medical history. She received the required prenatal tetanus toxoid vaccination, and her HIV test was negative. There was no history of maternal risk factors such as chorioamnionitis, premature or prolonged rupture of membranes, and maternal mastitis. She delivered in the hospital via spontaneous vaginal delivery, and the Apgar scores were 8/10 and 10/10 in the first and tenth minutes. Our index patient weighed 2.8 kg at birth. The neonate was exclusively breastfed. The mother and the babies were discharged on the same day of delivery.

On Day 3 postdelivery, the child was taken to the maternal and child health clinic for a routine follow‐up and was found to be well. However, on the eighth day after delivery, the child experienced intermittent fever, poor feeding, and irritability. The child’s symptoms were not improving, necessitating presentation to the neonatal unit of the hospital.

Physical examination revealed an acutely ill‐looking newborn with a temperature of 38°C, not pale, anicteric, acyanosed, well hydrated, and weighing 3 kg. Heart rate was 124 beats per minute, respiratory rate was 52 per minute, and oxygen saturation was 98%.

The abdomen was soft, moved with respiration, mildly distended, and showed no organomegaly. The umbilical region was erythematous and tender, with no apparent purulent discharge. The bowel sounds were normal. All other systemic findings were within normal limits.

The rapid diagnostic test for malaria was negative, and the hemoglobin concentration was 17.1 g/dL. We took a blood sample and requested a blood culture and an antibiotic susceptibility test. Due to the underequipped laboratory in our setting, we could not perform other relevant diagnostic tests, such as complete blood count and C‐reactive proteins, nor could we repeat the blood sample collection.

The neonate was diagnosed with bacteremia, presumed to be secondary to omphalitis. We commenced the child on the initial first‐line antibiotics as per the national guideline: intravenous (IV) ampicillin 50 mg/kg every 6 h and IV gentamicin 5 mg/kg daily. The child was placed on maintenance IV fluid of 10% dextrose saline and received expressed breast milk. We counseled the mother on proper body and hand hygiene and how to keep the umbilical area clean and dry.

The blood sample was incubated in the BACT/ALERT3D (Biomerieux Bact/Alert 3D: Marcy‐L’Etoile, France) blood culture machine and was detected as positive after 18 h. This was further subcultured onto 5% sheep blood (Oxoid CM0331B), MacConkey (Oxoid CM007), and chocolate (Oxoid CM0331B, Oxoid SR0090A, and Oxoid LP0053C) agar and incubated at 37°C overnight.

The agar plates showed a pure growth of large grey mucoid colonies with weak lactose fermentation on MacConkey media. A Gram stain revealed gram‐negative rods, followed by an analytical profile index (API) 20E test (BioMérieux 07584L), which showed an identification chart number of 1044172, consistent with the organism’s identity as *L. adecarboxylata*. The antibiotic susceptibility test demonstrated sensitivity to chloramphenicol, gentamicin, ciprofloxacin, cephalosporin, ampicillin, and amoxicillin–clavulanic acid but resistance to trimethoprim and tetracycline.

After 48 h of admission, the temperature dropped to 37.5°C, and the suckling reflex improved. Over the next 96 h, the baby maintained a temperature range of 36.2°C–36.7°C. The suckling reflex remained normal, and the inflammation around the umbilical cord stump resolved. The neonate was discharged home on the sixth day.

## 3. Discussion

*L. adecarboxylata* infections in neonates are rare, and the cases reported are commonly associated with preterm babies, nosocomial acquisition, long‐term antibiotic use, and invasive procedures, none of which is the case with our index neonate [10–13]. However, *L*. *adecarboxylata* infection has been previously reported in a healthy newborn [14].

*L. adecarboxylata* is common in the environment and exists as a commensal in the gut, which can facilitate opportunistic infections in vulnerable newborns [9]. The asymptomatic carriage of *L. adecarboxylata* on caregivers’ hands and unhygienic practices in newborn care, such as applying unrecommended substances like shea butter to the umbilicus, are the likely risk factors in our patient’s case. A newborn’s umbilical cord stump contains clotted blood vessels, making it a nutrient‐rich environment for microbial colonization. Poor care can give microbes direct access to the bloodstream, leading to an acute systemic infection [15].

Bacteremia is a common systemic complication of omphalitis and can progress to sepsis, which accounts for about 24% of neonatal deaths globally [16]. A similar case of *L. adecarboxylata*‐associated bacteremia, which ultimately progressed to sepsis, has been previously reported [10].

Omphalitis mainly occurs in rural areas of developing countries, with an incidence ranging from 2 to 7 cases per 100 live births [17]. According to the World Health Organization (WHO), omphalitis is diagnosed clinically by the presence of redness, swelling, and/or pus discharge at the umbilical cord stump [18]. In our case, the patient exhibited swelling, tenderness, redness, and differential warmth around the navel, accompanied by systemic symptoms indicative of bacteremia but no pus discharge.

A recent study by Josephine et al. found that the burden of omphalitis in rural areas of Central Uganda was 8.5% [19]. Another survey in Zambia showed that out of 1009 babies enrolled, 6.2% had omphalitis [20]. Among other causes, omphalitis mainly results from nonsterile birth environments, unhygienic care of the umbilicus, and unsanitary cultural practices [3, 18]. The traditional application of unrecommended substances such as shea butter, mustard oil, ghee, petroleum jelly, hair lotion, baby lotion, mud, ash, ginger, chewed rice, cow dung, and other harmful substances to the umbilicus due to cultural beliefs and healing purpose constitutes a significant factor in neonatal infections [3].

The WHO‐recommended umbilical cord care involves dry cord care in regions with low neonatal mortality and the daily use of 4% chlorhexidine to replace traditional substances like shea butter, especially in areas with high neonatal mortality [21]. Despite widespread access to information and advanced postnatal healthcare education, social barriers still hinder the adoption of recommended practices. Factors such as education level, birthplace, cultural beliefs, birth setting, and caregiver influence all play a role [3, 22]. This highlights the need for community‐wide health outreach beyond the hospital programs for mothers.

To the best of our knowledge, this is the first reported case of *L. adecarboxylata* infection in a neonate, presumed to be secondary to omphalitis, in The Gambia [23]. In low‐resource settings, there is a likelihood of *L. adecarboxylata* being misidentified as *E. coli* due to the similar metabolic and biochemical properties shared by both *Enterobacteriaceae* [24], thereby obscuring the true prevalence of *L. adecarboxylata* infections.

As demonstrated in our case and a similar one reported by Myers et al. [10], the infection generally resolves without complications when the recommended first‐line antimicrobials are administered promptly after diagnosis and managed properly, before it progresses to organ failure. In sub‐Saharan Africa, *L. adecarboxylata* has been found to lack genomic antibiotic resistance [25]. However, recent studies have identified carbapenem‐resistant strains [14, 26].

This report adds to the limited number of documented cases of *L. adecarboxylata* bacteremia in neonates, highlights the importance of ongoing awareness of proper umbilical cord care at the community level, and shows that early detection and management are vital for successful treatment.

**Nomenclature**
WHO: World Health Organization
IV: Intravenous

## Ethics Statement

The clinical care provided was in a clinical trial approved by the Gambian Government Ministry of Health and Medical Research Council Gambia Joint Ethics Committee (Reference: 1577, Date: 19 March 2018). The case was reported from a participant who was recruited as part of the Pneumococcal Vaccine Schedules (PVS) clinical trial. The trial lasted for five years, from 2018 to 2023. The ethical approval for the trial was obtained on March 19, 2018. This case was presented to the clinic in October 2023.

## Consent

Written informed consent was obtained from the patient’s parent for the publication of this case report. A copy of the written consent is available for review by the Editor‐in‐Chief of this journal.

## Disclosure

All authors read and approved the final version of the manuscript.

## Conflicts of Interest

The authors declare no conflicts of interest.

## Author Contributions

M.M., O.B., M.W., and R.S. provided the microbiology laboratory work results. M.B.O. wrote the first draft of the report. A.O.Y. assisted in writing the case report. B.M.W. and W.O.A. reviewed the first draft. I.O. and M.L.K. assisted in the patient’s management. I.O. and G.M. provided clinical work support, supervision, and writing of the case report manuscript.

## Funding

The authors used no funds in producing this manuscript.

## Data Availability

Data sharing is not applicable to this article.
